# Too Fat to Fit through the Door: First Evidence for Disturbed Body-Scaled Action in Anorexia Nervosa during Locomotion

**DOI:** 10.1371/journal.pone.0064602

**Published:** 2013-05-29

**Authors:** Anouk Keizer, Monique A. M. Smeets, H. Chris Dijkerman, Siarhei A. Uzunbajakau, Annemarie van Elburg, Albert Postma

**Affiliations:** 1 Experimental Psychology/Helmholtz Institute, Utrecht University, Utrecht, The Netherlands; 2 Faculty of Social and Behavioural Sciences, Utrecht University, Utrecht, The Netherlands; 3 Department of Neurology, University Medical Centre Utrecht, Utrecht, The Netherlands; 4 Eating Disorders Rintveld, Altrecht, Zeist, The Netherlands; Royal Holloway, University of London, United Kingdom

## Abstract

To date, research on the disturbed experience of body size in Anorexia Nervosa (AN) mainly focused on the conscious perceptual level (i.e. *body image*). Here we investigated whether these disturbances extend to *body schema*: an unconscious, action-related representation of the body. AN patients (n = 19) and healthy controls (HC; n = 20) were compared on body-scaled action. Participants walked through door-like openings varying in width while performing a diversion task. AN patients and HC differed in the largest opening width for which they started rotating their shoulders to fit through. AN patients started rotating for openings 40% wider than their own shoulders, while HC started rotating for apertures only 25% wider than their shoulders. The results imply abnormalities in AN even at the level of the unconscious, action oriented body schema. Body representation disturbances in AN are thus more pervasive than previously assumed: They do not only affect (conscious) cognition and perception, but (unconscious) actions as well.

## Introduction

Body representation disturbances are considered a key-symptom of Anorexia Nervosa (AN) [1], [2], [3], [4], [5], and found to be persistent, even after otherwise successful treatment [6], [7]. The current study shows that body representation disturbances in AN are even more pervasive than previously assumed, and go beyond a distorted (mental) *image* of the body. Specifically, we show here that action-guidance in AN patients is based on enlarged body size input. Thus, AN patients do not only think that they are fat, and perceive themselves as fat, even their motor behaviour is consistent with such beliefs and perceptions, as patients were found to walk through a door-like opening as if they were fatter than they actually were. Our study is the first that directly targets action-related body representation disturbances in AN.

Previous research on the disturbed experience of body size and shape in AN has focused mostly on body related deviancies in cognition/affect and (body size) perception. Typically AN patients show high levels of body dissatisfaction (e.g. [8], [9]) and visually perceive/imagine their body as fatter than it is (e.g. [10], [11], [12]). To ensure optimal treatment, we believe it is crucial to gain insight into all facets of body representation disturbances in AN. For example, recently it was found that AN patients’ tactile perception is altered as well: Patients perceived tactile stimuli on their skin as further apart than they actually were [13], [14]. However, no studies have yet directly addressed body representation disturbances in AN beyond perceptual processing, and focused on the possibility that body representation disturbances could extend to more unconscious, *action*-related, aspects of body representation. This is surprising, as body-scaled action may be affected by an inappropriate mental representation of body size.

Body-scaled action is closely linked to the so-called *body schema*. Traditionally, in literature on body representation a distinction is made between body image and body schema [15]. We adopt here the definition of body schema as an unconscious, sensorimotor, representation of the body that is invoked in action [15], [16], [17], [18], [19]. It is believed to store, and constantly update, information on e.g. the exact location of the body and its parts in space, and on how separate parts of the body form a coherent whole (e.g. [19]). Body schema aids action guidance by providing information on where the body is in space, and given its size, which actions it affords relative to obstacles in the environment [15], [20]. Body image on the other hand is its conscious counterpart that is mainly used in body-size related perception and (affective) cognition [15], [16], [17], [18]. Note however that different researchers adopt different definitions of body schema (see e.g. [15]). Body schema may be used to refer to mainly postural information and somatic input concerning the location of a tactile stimulus on the skin [21], but is has also been be used as an umbrella term which encompasses body form representations as well, instead of only action-related processing [22].

In the present study we measured body-scaled action by having participants walk through door-like openings varying in width. To do this safely and efficiently, participants need to access information on the location and size of their body relative to external objects [23]. Constant updating and processing of this information is suggested to take place outside awareness, which enables individuals to perform actions like navigating past obstacles/other individuals relatively effortlessly [24]. In general, Individuals successfully rotate their body as soon as an open space is smaller than the widest frontal dimension of their body, without investing much conscious effort.

The possibility of body schema disturbances in AN has been suggested previously [25], [26], [27]. For example, a motor imagery study, in which participants *imagined* walking through a projected aperture, showed that AN patients indicated they would rotate their shoulders for relatively larger apertures than healthy controls [25]. This was interpreted as indicative of a body schema disturbance [25]. It should be noted that participants did not actually perform the action of walking in this study. Importantly, in this previous work [25] it cannot be ruled out that AN patients made a conscious decision about having to rotate their shoulders or not. Such a conscious decision might reflect AN patients’ emotional/cognitive state towards their body; i.e. “I am fat, thus I will not fit through this opening without rotating”. A direct assessment of the body schema in action at a more implicit level has not been made yet in AN. In the current study we therefore assessed actual *walking* through an aperture, while having participants focus their attention on a diversion task, and not on their rotating behavior, ensuring a relatively unconscious measurement of body schema. As AN patients are overly aware of their body (size), we aimed to distract them from how they moved their body in space as much as possible, and presented the apertures as “panels” with no apparent significance.

Previous findings with healthy participants have shown that the width of an opening for which participants start to rotate their body in order to fit through can be expressed as critical aperture (A) to shoulder (S) ratio (A/S_crit_) [23]. For example, with an individual shoulder width of 40 cm, and rotation starting at an aperture width of 50 cm, A/S_crit_ = 1.25 (50/40). A/S_crit_ was found to be constant across healthy participants, independent of body height or width [23]. In accordance with motor imagery findings [25], [26] we hypothesized that AN patients would show a higher A/S_crit_ than healthy controls, i.e. that AN patients would start to rotate their shoulders at relatively larger openings than healthy controls would.

## Methods

### Ethics Statement

The current study was approved by two independent ethics committees (Medical Ethical Committee University Medical Centre Utrecht and the Committee Scientific Research of Altrecht Rintveld Eating Disorders). The study adhered to the tenets of the Declaration of Helsinki. Each participant received an information letter about the study and the study procedures. At the start of the experiment the procedures were verbally explained by the researcher, after which written informed consent from the participant was obtained.

### Participants

Thirty-nine females, over 18 years of age, and without physical conditions preventing them from walking, participated: 19 patients (13 AN patients and 6 Eating Disorder Not Otherwise Specified patients (EDNOS) [1], and 20 healthy undergraduate students. Presence of an eating disorder was excluded in healthy controls (HC) using the Eating Disorder Examination Questionnaire (EDE-Q) [28]. AN and EDNOS patients were diagnosed with the Eating Disorder Examination (EDE) [29] and a psychiatric interview. Patients received treatment as usual ranging from daily to weekly sessions at an eating disorder clinic. Treatment aimed at weight gain, and due to increased BMI at the time of the study, some patients’ initial AN diagnosis changed to EDNOS. However, it was found that EDNOS is similar to AN [30], [31], and that a combined AN/EDNOS group is homogeneous (e.g. [32]). Indeed, here AN and EDNOS patients did not have different scores on any of the tasks (see results section). Therefore we will refer to the AN/EDNOS group as the AN patient group or patient group.

AN patients and HC did not differ in age, t(25.01) = −2.97, p = .127 (M_AN_ 23.68, SD = 4.62; M_HC_ = 21.90, SD = 2.13). HC had a higher BMI than AN patients, t(36.96) = −2.98, p = .001 (M_AN_ = 18.32, SD = 2.69; M_HC_ = 21.00, SD = 1.55). Disease duration for AN patients ranged from 2 to 50 months (M = 15.00, SD = 14.04). Note that patients may have received treatment elsewhere as well.

### Materials and Procedure

The experiment started with measuring body height, shoulder height and the horizontal width between the shoulders, waist, elbows, hips, and knees. Participants were told infrared cameras hanging from the ceiling would record reflective markers that would be placed on their body, from which their movements throughout the experiment could be inferred, and that the body measurements were necessary to calibrate these cameras. In reality only measures of shoulder height and width were used for this purpose. After completion of the aperture task, perceptual body image was assessed by asking participants to estimate their body width and their shoulder width, to verify perceptual body size representation disturbances in the AN group.

### Aperture Task

**Figure 1:**
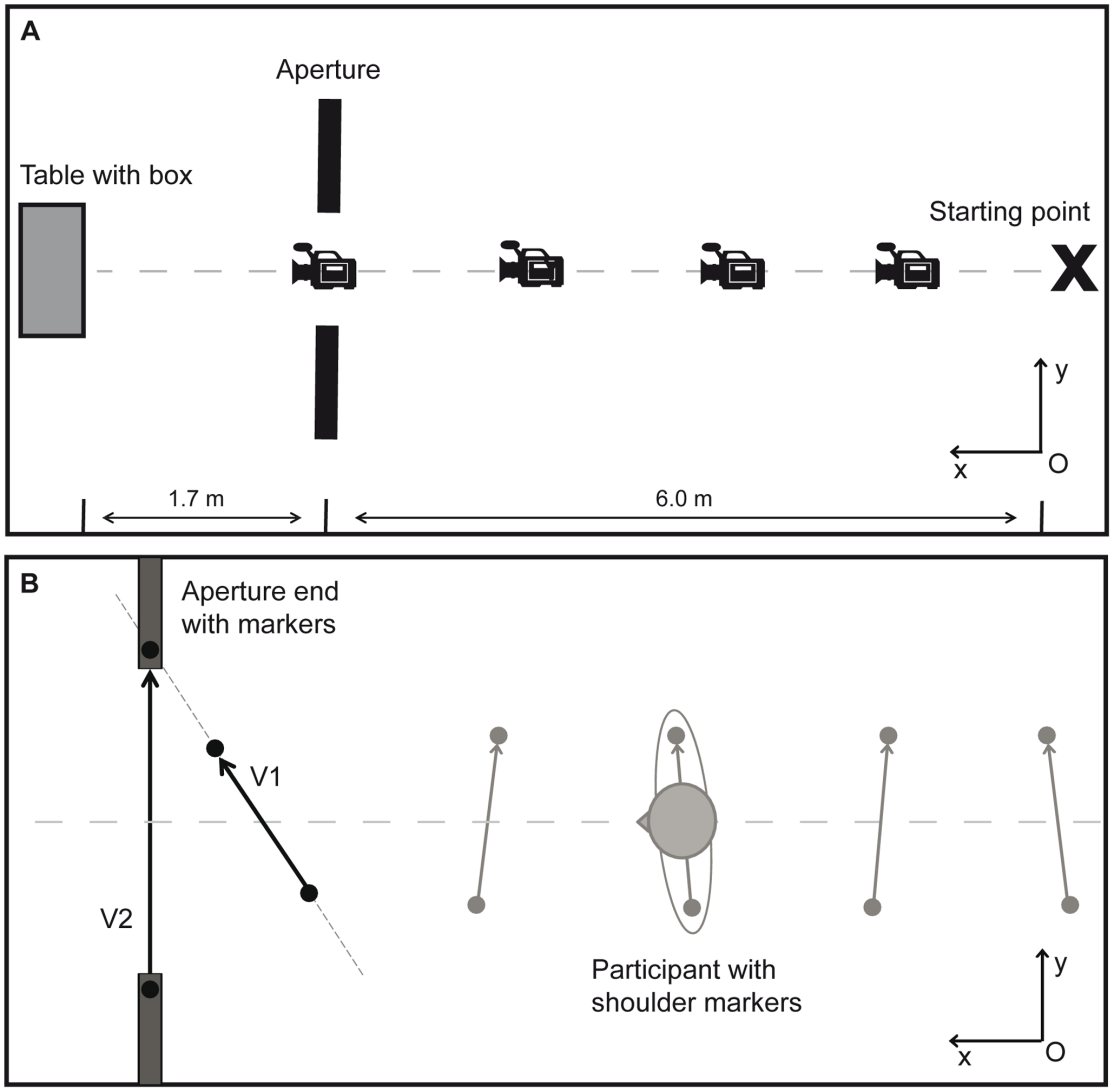
Set up of the aperture task (Panel A) and calculation of shoulder rotation (Panel B). Schematic set up of the aperture task (Panel A) and calculation of shoulder rotation (Panel B). Panel A: “Starting point” refers to the marked spot on the floor where each participant stood on at the beginning of a trial; “Aperture” refers to the wooden partitions forming an opening that varied in size; “Table with box” refers to the endpoint of each trial, on which a box was placed that was used as part of the haptic memory distracter task. Panel B: V1 and V2 refer to the vectors calculated in the global coordinate system. The amount of shoulder rotation was defined by calculating the angle between V1 and V2.

Each trial (see Figure 1) started with the participant standing on the starting point. Participants walked 7.7 meters towards a table placed behind an aperture. Six meters from the starting point the participants walked through the aperture, which consisted of two grey movable wooden partitions (1.97×1.23 m). After each trial, participants waited behind a screen while the experimenter prepared the set-up for the following trial. In total 36 trials were completed, consisting of 12 different aperture widths that were each presented three times. Aperture width was based on the actual shoulder width of the participants, and ranged from A/S = 0.9 to A/S = 2.0, in steps of A/S = 0.1. Using a tailored MATLAB® (The MathWorks®) routine, aperture width was determined, and the trials were presented in a randomized order.

Four Optitrack™ cameras type V100:R1 recorded (100 Hz) two passive reflective markers (12.7×12.7 cm) that were placed on the shoulders of the participants (on top of the humerus) and on the edges of the partitions. The angle of the shoulders relative to the partitions was calculated for each trial using a tailored MATLAB® routine. In the calculations, a global two-dimensional coordinate system was assumed to coincide with the floor of the room. The origin of the global coordinate system was assumed to be located at the right bottom corner of the field of view of the first camera (denoted as “O” in Figure 1). Coordinates of all markers in the global coordinate system were calculated for each recorded frame based on positions of the markers in the fields of view of the cameras and height-dependent scaling coefficients. The scaling coefficients were determined by recording a system of markers located on the floor of the room and by measuring height of the markers on the participants’ shoulders and that of the markers on the partitions. Subsequently, two vectors (denoted as V1 and V2 in Figure 1) were calculated in the global coordinate system. Vector V1 points from the left to the right shoulder marker. Similarly, vector V2 points from the left to the right partition marker. The angle of the shoulders relative to the partitions was calculated as an angle between vectors V1 and V2 (see Figure 1). The coordinates of the middle point between the markers on the shoulders of the participant, as well as the markers on the partitions, were considered as a measure of the position of the participant and the aperture.

When the angle of the shoulders was larger than the natural sway in between one meter before and half a meter behind the aperture, a participant was said to have rotated her body in order to fit though the opening. Natural sway was defined as the maximum angle of shoulder rotation from starting point till one meter before the aperture. A/S_crit_ was defined as the widest aperture for which a participant rotated her shoulders at least two out of three trials. Mean walking speed in km/h was determined over all trials. For rotation trials the maximum angle of shoulder rotation and onset of shoulder rotation were calculated. Onset of shoulder rotation was defined as the distance (in cm) from the aperture at which shoulder rotation was first larger than the natural sway. For both variables we were mainly interested in performance at A/S = 0.9, the smallest aperture width for which each participant had to rotate, otherwise she would not be able to fit through it, and performance at A/S_crit._

Participants were unaware of the actual purpose of the study (i.e. measuring shoulder rotation while crossing apertures), and were led to believe the experiment focused on haptic memory. At the start of each walking trial participants explored a relief structure (10×2.5 cm; based on [33]) and were instructed to memorize the pattern. At the end of the walking trial, participants explored two relief structures placed in a box at the table located at the end of the room, and choose which one was most similar to the one explored at the beginning of the trial. Participants were told the walking enabled the researchers to investigate whether performing a different action (exploration with hands vs. walking) would influence haptic memory. Apertures were referred to as panels and only mentioned in the walking instructions: “*Walk in a straight line towards the table, using the midpoint between the panels as a guide. The panels may change location during the experiment, e.g. from left to right, or front to back. You are assigned the condition in which they move from left to right. Please walk as natural as possible, you can walk and move however you prefer, as long as you walk in a straight line towards the table.*” The experiment was thus presented as investigating haptic memory by analyzing how participants explored the haptic patterns, and whether exploration could be influenced by performing a different action (walking) in between the first (start of walking trial) and second (end of walking trial) exploration. To make it more convincing that explorative hand movements were of interest, fake markers were placed on the participants’ hands, wrists, and elbows. At the end of the aperture task participants completed a questionnaire assessing the study objectives in which we explicitly asked what they thought the aim of the study was, and whether they noticed anything about the apertures (“panels”), the table in the room, and the box containing the haptic patterns. All participants indicated that they thought the main aim of the experiment was related to haptic memory, and described strategies for exploring and memorizing the different structures. None of the participants guessed that we were actually interested in measuring shoulder rotation, neither did they notice anything other than the panels moving across trials about the apertures.

### Body Image

Perceptual body image was assessed after the Aperture task by asking participants to estimate their body width as well as their shoulder width specifically. To assess general body size participants stood on the starting point with aperture width set at A/S = 2.0. The experimenter pushed the partitions closer together and the participant indicated when she would be able to fit exactly in the space between the apertures. This was repeated while the aperture was closed and the experimenter pulled the partitions apart. The average width of the pushing and pulling condition was taken as a measure of body size estimation. To assess shoulder width specifically, participants were instructed to draw a vertical line on a whiteboard which represented the width of their shoulders. We choose a vertical line in this task, because when drawing a horizontal line, participants could have easily used their own body as a reference while drawing.

## Results

### Aperture Task

AN patients had a significantly higher A/S_crit_ than HC, t(37) = 3.84, p<.001 (M_AN_ = 1.40, SD = 0.15; M_HC_ = 1.25, SD = 0.09), implying that AN patients rotated their shoulders for relatively larger apertures than HC. A/S_crit_ did not correlate with BMI, r_AN_ = .18, p = .466; r_HC_ = .08, p = .724, disease duration, r_AN_ = .07, p = .800, or walking speed, r = −.14, p = .416. Independent samples *t*-tests showed that AN patients and HC differed on walking speed over all trials, t(35) = −2.67, p = .011 (M_AN_ = 5.98, SD = 0.53; M_HC_ = 6.41, SD = 0.47). The groups did not differ on mean maximum angle of rotation at A/S = 0.9 and A/S_trials_ trials, and onset of rotation at A/S = 0.9 and A/Sc_rit_ trials (see Table 1).

**Table 1:** Results for the aperture task, shoulder width measurement, and shoulder width estimation by group.

	AN (N = 19)		HC (N = 20)			
	M (SD)		M (SD)		t(37)	p
A/S_crit_	1.40	(0.15)	1.25	(0.09)	3.84	<.001
Walking speed (km/h)	5.98	(0.53)	6.41	(0.47)	**−2.67	.011
Max rotation A/S = 0.9 (in °)	72.00	(12.10)	68.05	(13.33)	*0.96	.345
Max rotation A/S_crit_ (in °)	32.37	(18.55)	31.86	(18.08)	0.09	.932
Onset rotation A/S = 0.9 (in cm from aperture)	75.72	(24.03)	76.08	(29.22)	*−0.04	.967
Onset rotation A/Sc_rit_ (in cm from aperture)	61.39	(19.33)	48.80	(24.60)	1.77	.085
Absolute aperture width at A/S_crit_ (in cm)	51.91	(7.86)	49.42	(4.20)	1.27	.213
Shoulder width (in cm)	37.03	(2.82)	39.55	(2.16)	−3.15	.003
Estimated shoulder width (in cm)	47.08	(9.66)	43.68	(8.47)	−0.30	.767
Overestimation shoulder width (in %)	27.85	(28.03)	10.68	(22.42)	2.12	.041

*Note*. * refers to a df of 36 due to missing data in the HC group (n = 1), **refers to a df of 35 due to missing data in the HC group (n = 1) and an outlier in the AN group (n = 1).

Although we grouped AN and EDNOS patients together in the current experiment, we checked for differences between these groups, but found none. Most importantly, there were no significant differences between AN and EDNOS patients for A/S_crit_, t(17) = −0.65, p = .523 (M_AN_ = 1.39, SD = 0.16; M_EDNOS_ = 1.43, SD = 0.15). AN and EDNOS patients also did not differ with regard to other variables associated with the aperture task and locomotion: Walking speed in km/h, t(17) = 0.19, p = .848 (M_AN_ = 6.30, SD = 1.62; M_EDNOS_ = 6.17, SD = 0.49); maximum rotation in degrees at A/S = 0.9, t(17) = 0.13, p = 901 (M_AN_ = 72.25, SD = 13.43; M_EDNOS_ = 71.47, SD = 9.66); maximum rotation at A/S_crit_ in degrees, t(17) = 0.57, p = .579 (M_AN_ = 34.03, SD = 21.13; M_EDNOS_ = 28.76, SD = 11.97); onset of rotation at A/S = 0.9 in cm from aperture, t(17) = −0.30, p = .770 (M_AN_ = 74.57, SD = 15.60; M_EDNOS_ = 78.20, SD = 38.51); onset of rotation at A/S_crit_ in cm from aperture, t(17) = 0.65, p = .524 (M_AN_ = 63.38, SD = 19.08; M_EDNOS_ = 57.07, SD = 20.96); and absolute aperture width at A/S_crit_ in cm t(17) = .19, p = .848 (M_AN_ = 50.73, SD = 8.00; M_EDNOS_ = 54.48, SD = 6.86).

Taken together, the patient group and HC group differed in the opening size for which they started rotating, but behaved quite similar on other aspects of locomotion.

### Body Image

Note that all analyses reported below were, unless stated otherwise, independent samples *t*-tests. AN patients had significantly smaller shoulders than HC, t(37) = −3.15, p = .003 (M_AN_ = 37.03 cm, SD = 2.82; M_HC_ = 39.55, SD = 2.16), see Table 1. To take this difference in actual shoulder width into account we calculated the percentage of overestimation as 100*(estimated-actual)/actual (see [34]).

The results from the general body size estimation task (indicating body width by adjusting the aperture) showed a between-group difference, with AN patients showing significantly higher percentages of overestimation of their body size than HC, t(37) = 5.02, p<.001 (M_AN_ = 46.29%, SD = 15.08; M_HC_ = 23.54%, SD = 13.23). One sample *t*-tests showed that both AN patients and HC significantly overestimated their body size, t_AN_(18) = 13.38, p<.001; t_HC_(19) = 7.96, p<.001. Percentage of body size overestimation correlated with disease duration, r_AN_ = −.54, p = .017, but not with BMI, r_AN_ = .16, p = .503; r_HC_ = −.11, p = .631. When comparing the absolute aperture width (in cm) for which participants started rotating their body (i.e. width of the aperture in cm at A/S_crit_) with the absolute estimation of body size (in cm) using paired samples *t*-tests we did not find differences for the AN patients, t_AN_(18) = 1.01, p = .325 nor HC, t_HC_(19) = −.39, p = .700.

More importantly, in the body image task in which shoulder width was specifically estimated by drawing a line on a whiteboard, AN patients showed higher percentages of overestimation than HC, t(37) = 2.12, p = .041 (M_AN_ = 27.85%, SD = 28.03; M_HC_ = 10.68%, SD = 22.42), see Table 1. One sample *t*-tests showed that both groups overestimated their shoulder width, t_AN_(18) = 4.33, p<.001; t_HC_(19) = 2.13, p = .047. Size estimation of shoulder width (body image measure) did not correlate with A/S_crit_ (body schema measure), r_AN_ = −.35, p = .146; r_HC_ = .07, p = 786.For AN patients, but not HC, shoulder width estimation correlated with BMI, r_AN_ = −.54, p = .017; r_HC_ = −.26, p = .268. No correlation between estimation of shoulder width and disease duration was found, r_AN_ = −.23, p = .352.

A/S_crit_ from the aperture task was based on the largest aperture width for which participants rotated at least two times (A) divided by the participants’ shoulder width (S). AN patients and HC did not differ on the absolute value of A in cm, t(37) = 1.27, p = .213 (M_AN_ = 51.91 cm, SD = 7.68; M_HC_ = 49.42 cm, SD = 4.20), see Table 1. Instead of using S to calculate A/S_crit_, A/S_estimated_crit_ can be determined as well. A/S_estimated_crit_ refers to the largest aperture width for which participants rotated at least two times (A) divided by the participants’ absolute *estimate* of her shoulder width in cm in the body image task (S_estimated_). A/S_estimated_crit_ is thus a parameter indicative of what participants’ ratio would be if their shoulders were in reality as wide as they estimated them to be. AN patients and HC did not differ in A/S_estimated_crit_, t(37) = −0.30, p = .767 (M_AN_ = 1.14, SD = 0.28; M_HC_ = 1.17, SD = 0.22). In addition, when comparing HC’s A/S_crit_ with AN’s A/S_estimated_crit_ there were no significant differences, t(21.46) = −.1.59, p = .127 (M_AN_ = 1.14, SD = 0.28; M_HC_ = 1.25, SD = 0.09). In other words, if AN patients’ shoulders were to be as wide as they estimated them to be, their performance on the aperture task would be the same as that of HC.

Taken together these results show that both AN patients and HC overestimate their general body size as well as their shoulder width specifically, although the percentage of overestimation was higher for AN patients. Based on estimated shoulder width, performance on the aperture task would have been comparable to normal in AN patients.

## Discussion

Previous research on body representation disturbances in AN has mainly focused on perceptual body image disturbances (see e.g. [8], [11]), while studies on action-related components of body representation are relatively absent. To ensure optimal treatment and understanding of the disturbed experience of body size in AN, it is important to know the extent of this disturbance, i.e. is it limited to the perceptual body image, or does it extend to action-related body schema? Therefore, the present study assessed potential disturbances in body-scaled action in AN patients. Body-scaled action was measured with an aperture task (see e.g. [23]), with as main parameter of interest the critical aperture (A) to shoulder (S) ratio (A/S_crit_), i.e. the relative width of the aperture for which participants started to rotate their shoulders in order to fit through [23]. The results clearly showed that AN patients had a higher A/S_crit_ than HC, indicating that AN patients rotated their shoulders for relatively larger openings than HC (A/S_crit_ 1.40 vs 1.25).

The current results are a first demonstration of AN patients having a disturbance in body-scaled action, as their adaptive postural changes during body-scaled action appeared to be based on an enlarged representation of body size. The results further revealed that AN patients, compared to HC, showed a higher percentage of overestimation of their shoulder width in a perceptual body image task in which they drew a line representing the distance between their shoulders. In addition, we calculated A/S_estimated_crit_ based on the absolute aperture opening for which participants started rotating in the aperture task (A) and their absolute *estimated* shoulder width (S_estimated_). Interestingly, these results suggest that if AN patients’ shoulders were as wide as they estimated them to be, they would perform equal to HC on body-scaled action. This implies that stored information on body size is disturbed in AN, which in turn affects perception-related body image as well as action-related body schema representations.

The present study provides two critical extensions compared to previous work. First, in contrast to the study by Guardia et al. [25], we measured real action performance. Second, during the aperture task participants were unaware of the aim of the study, as they focused their attention on memorizing haptic patterns. Although AN patients are chronically aware of their body (size) [1], it is unlikely that here their decision to either rotate while crossing an aperture, or walk straight through, was consciously influenced by negative affective/emotional top-down input related to their body size. When explaining the procedures and during the experiment participants were led to believe the objective of the study was assessing haptic memory. Great care was taken to minimize the apparent significance of the apertures, which were referred to as “panels” and mentioned as little as possible. In addition, would AN patients have made such conscious rotating decisions, this would likely have affected other typical locomotor adjustments associated with crossing an aperture such as onset of rotation or amount of rotation (e.g. [35], [36]). We did not find differences on these variables between AN patients and HC. The results did show a small difference in walking speed, with AN patients walking slightly (less than half km/h) slower than HC. As AN patients’ A/S_crit_ was higher than that of HC, AN patients rotated on *more* trials than HC during the experiment. Previous research showed that trials on which participants rotate their shoulder result in decreased walking speed, compared to non-rotation trials [36], which is likely to explain reduced walking speed in the AN group. Overall, it appears that during action motor-programs process information accurately, but the body size related *input* they receive is not in accordance with actual body dimensions of the AN patients.

As also suggested by e.g. Guardia et al. [25], [26] body schema disturbances in AN may be related to a failure to update body-schema to new (smaller) body dimensions after initial weight-loss. Our patient group consisted of a combination of AN and EDNOS patients. Indeed, the AN patients in our sample have lost weight over time, and their body schema might not be updated to their now (extremely) small body size. In contrast, the EDNOS patients included in our sample entered treatment diagnosed with AN (i.e. underweight), but gained weight during treatment, resulting in no longer fulfilling the diagnostic criteria for AN due to a healthy BMI and body size at the time of the experiment. In other words, the EDNOS patients did not *have* smaller body dimensions that their body schema should have been updated to. Still their A/S_crit_ was found to be higher than that of HC, and did not differ from AN patients’ A/S_crit_. This deems failure to update body schema to smaller body dimensions an unlikely explanation for patients’ higher A/S_crit_. Future studies investigating body-scaled action in healthy participants who lost (or gained) a significant amount of weight could shed more light on the issue of updating body schema to changed body dimensions and its possible influence on body-scaled action.

The present study further raises questions regarding the relation between disturbances in body-size related perception and action. Should AN patients’ altered body-scaled action be viewed as separate from previously identified disturbances in body image (e.g. [11], [12], [14]), could either one be causally linked to the other, or is perhaps the relation between body image and body schema distortions more dynamic? Literature on body representations does not offer a conclusive answer on this matter. Although the two concepts – body image and body schema – are traditionally viewed as two separate representations (see e.g. [16], [17], [18]), it has also been suggested that perception and action cannot be viewed as completely independent (see e.g. [15], [20]). In other words, we might be able to distinguish between different body representations that play different roles in different situations, but they nevertheless interact and overlap with each other, as encoding of bodily information depends on task demands, e.g. which information is required in a certain context [15].

It is beyond the scope of this paper to resolve the body image vs body schema issue. However, the current study does imply that body size related distortions in AN are more pervasive than previously assumed, and that they affect not only perception, but action as well, which has important implications for treatment. Usually the aim of interventions targeting the disturbed experience of the body in AN is changing cognitions and perception of body size. Several studies have shown that such approaches are not always efficient, as after otherwise successful treatment, body-size issues remain [7]. This may be because current interventions mainly attempt to change aspects of body image, and not of body schema. It could therefore be relevant to design a treatment strategy in which action related responses are targeted as well. Interventions focusing on sensorimotor feedback, for example prism adaptation, were already found to be successful in influencing action as well as perceptual representations in hemispatial neglect [37] and complex regional pain syndrome patients [38].

In sum the present study suggests that when crossing an aperture AN patients used, on an implicit level, body size information congruent with how they perceive themselves, instead of their actual (smaller) body dimensions. It thus appears that for AN patients experiencing their body as fat goes beyond thinking and perceiving themselves in such a way, it is even reflected in how they move around in the world. This indicates that the disturbed experience of body size in AN is more pervasive than previously assumed.
